# Nickel-induced labial angioedema in a pediatric patient with orthodontic braces: a case report

**DOI:** 10.1186/s13052-024-01833-4

**Published:** 2025-01-05

**Authors:** Fabrizio Leone, Alessandra Gori, Bianca Laura Cinicola, Giorgio Colletti, Elia Pignataro, Martina Capponi, Giulia Brindisi, Caterina Anania, Anna Maria Zicari

**Affiliations:** 1 Department of Maternal Infantile and Urological Sciences, Sapienza University of Rome, Rome, 00161 Italy; 2 Department of Translational and Precision Medicine, Sapienza University of Rome, Rome, 00161 Italy; 3 Department of Experimental Medicine, Sapienza University of Rome, Rome, 00161 Italy

**Keywords:** Nickel Allergy, Angioedema, Dental braces, Contact urticaria, Case report

## Abstract

**Background:**

Angioedema is a condition marked by sudden, intense swelling of the subcutaneous and submucosal tissues, typically associated with hypersensitivity reactions, genetic mutations, or reactions to medications. It can also result from contact with allergens such as nickel, leading to dermatitis.

**Case presentation:**

: A 12-year-old girl presented at our Pediatric Immunology and Allergology service with recurrent labial angioedema for over a year, linked to the consumption of legumes and tomatoes, and following the use of a metal flute. Despite a nickel-positive patch test and subsequent avoidance of nickel, her symptoms persisted. Further investigations to rule out other causes of angioedema were unproductive. It was later discovered that she had been wearing a nickel-containing orthodontic device applied a year earlier. The removal of this orthodontic device led to a cessation of the angioedema episodes, highlighting nickel as the likely trigger.

**Conclusions:**

This case underscores the importance of considering prolonged nickel exposure from dental devices as a potential cause of angioedema. For patients predisposed to nickel hypersensitivity, using nickel-free alternatives such as ceramic for orthodontic appliances is crucial. Additionally, comprehensive allergen screening, including latex testing, should be conducted before the placement of such devices to prevent similar adverse reactions.

## Background

Although both are reactions to external substances, Angioedema (AE) and contact dermatitis (CT) have distinct immunological mechanisms. This distinction is crucial in the clinical arena, especially in the complex interaction between these conditions and external allergens, such as nickel (Ni), commonly encountered in everyday materials, including orthodontic devices.

AE is primarily associated with Type I hypersensitivity reactions, involving the release of histamine and other chemicals from mast cells and basophils, or with deficiencies or dysfunctions of the C1 Inhibitor (C1INH), leading to uncontrolled release of bradykinin [1, 2]. These mechanisms result in deep tissue swelling, often without itchiness, manifesting rapidly after exposure to the allergen [2]. The complexity increases with angioedema resulting from drug reactions, such as those caused by ACE inhibitors, which decrease the breakdown of bradykinin or hereditary conditions involving specific mutations [1, 2].

In contrast, CT falls under the Type IV hypersensitivity category, which is characterized by a delayed immune response [3, 4]. This process begins when allergens such as nickel make contact with the skin, are processed by antigen-presenting cells (APCs), and are subsequently recognized by T cells, leading to inflammation, rash, and itching that develops over days. The primary mediators in CT are T lymphocytes and cytokines, affecting the epidermal layers of the skin, as opposed to the deeper, subcutaneous and submucosal tissues affected in angioedema [3, 4]. The main differences between these two entities are reported in Table 1.

**Table 1:** Comparative overview of Angioedema and Contact Dermatitis hypersensitivity reactions

	Angioedema	Contact dermatitis
**Type of Hypersensitivity**	**Type I Hypersensitivity**: associated with IgE-mediated mast cell activation **HAE (due to C1INH deficiency)**: includes HAE-type 1 (deficiency) or type 2 (malfunction) of C1 INH. It causes the uncontrolled release of BK. **Drug-Induced AE**: Some drugs (e.g. ACE inhibitors) can cause AE by reducing the levels of degraded BK. **Specific Mutations**: HAE-FXII XII, HAE-Plasminogen, HAE-Angiopoietin-1, HAE-Kininogen, HAE-1 Myoferlin, HAE-HS3ST, and unknown (idiopathic) HAE.	**Type IV Hypersensitivity**: delayed hypersensitivity reaction. When an allergen (such as nickel) comes into contact with the skin, it is processed by antigen-presenting cells and presented to T cells that mediate the immune response.
**Main Mediators**	Histamine, BK	T lymphocytes, Cytokines (e.g. IL-2, IL-4, IL-5, IFN-γ, TNF-α and IL-17)
**Localization**	Deeper tissues (subcutaneous and submucosal)	Epidermal layers of the skin
**Timing**	Immediate for Type I, Delayed for HAE	Delayed (hours or day)
**Symptoms**	Deep swelling, often without itchiness	Itching, redness, blisters/desquamation

HAE: Hereditary Angioedema; C1INH: C1-Inhibitor; BK: bradykinin; HS3ST: heparan sulfate-glucosamine 3-O-sulfotransferase 6 gene; IL-2: interleukin 2; IL-4: interleukin 4; IL-5; interleukin 5; IFN-γ: Interferon gamma; TNF-α: Tumor Necrosis Factor Alpha; IL-17: interleukin 17

This case report presents a scenario in which prolonged exposure to nickel from an orthodontic device triggers angioedema, a condition typically associated with more acute allergic reactions or genetic factors rather than delayed-type hypersensitivity reactions like contact dermatitis.

## Case presentation

**Fig. 1:**
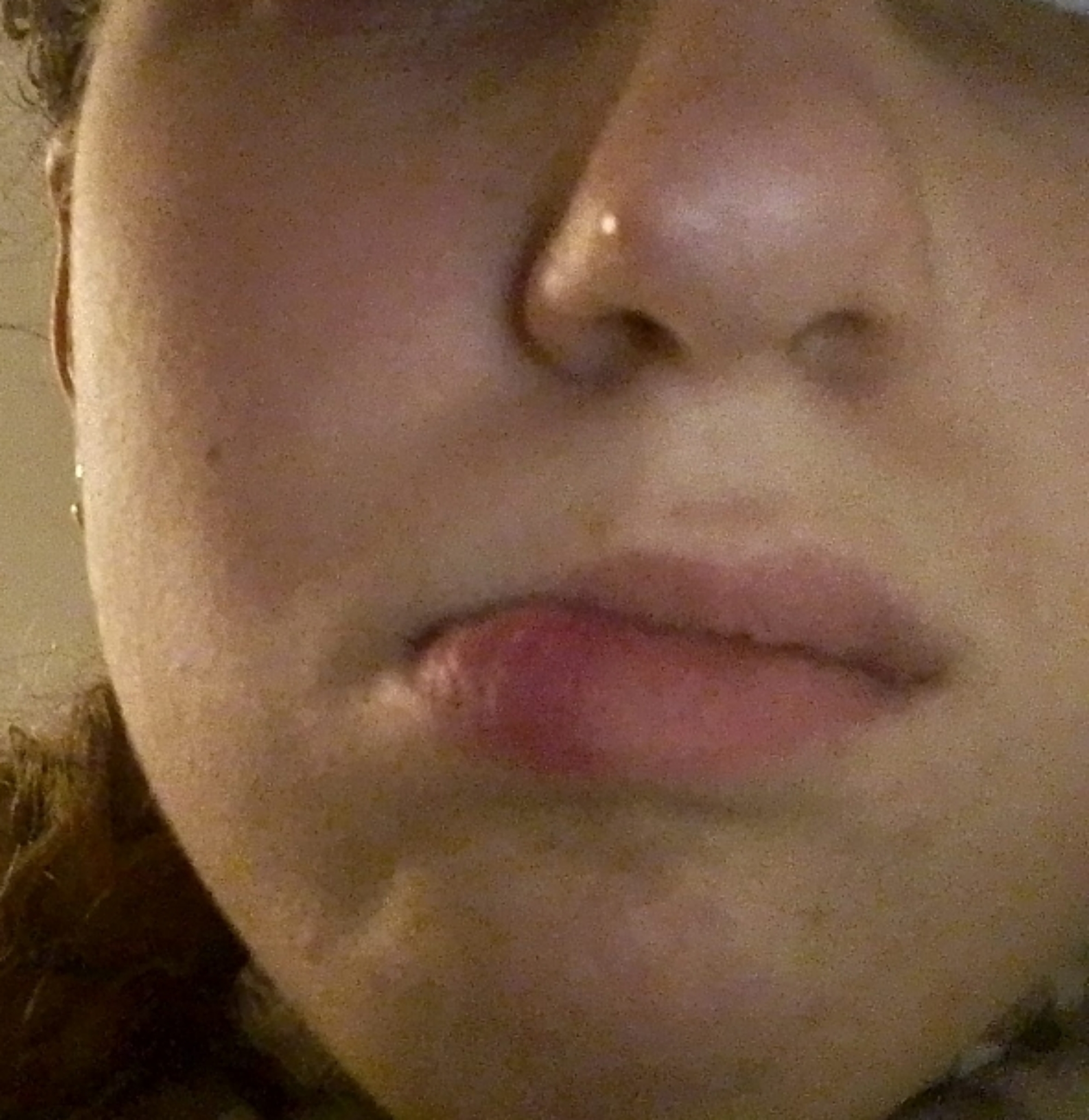
The photo represents an episode of the patient’s labial edema

The patient is a 12-year-old girl with a relevant medical history of episodes of labial and lingual angioedema (Fig. 1), mainly triggered by the ingestion of specific foods, including chocolate, lentils, nuts, cooked ham, clams, mussels, and certain types of tomato sauce. Additionally, she experienced similar episodes while playing a metal flute, characterized by swelling of the lips and tongue accompanied by limb itching. A switch to a wooden flute resulted in symptom improvement. Complete resolution of symptoms was achieved within 2–3 days with oral betamethasone. Her family history is notable for nickel allergy in her mother and maternal and paternal aunts. Despite the family history of nickel allergy, no one in the family has ever had recurrent episodes of angioedema. She does not have a history of atopic dermatitis, ocular or rhinitis symptoms, or asthma. During episodes of labial angioedema, she had a single-time mild respiratory failure resolved after taking betamethasone; she never had abdominal pain or other associated symptoms.

Patch tests confirmed a significant allergy to nickel sulfate (+++), along with positive reactions to paraben mix (++) and methyl(chloro)-isothiazolinone (+). Routine blood tests, including complete blood count, liver function tests, iron panel, thyroid function tests, and screening for celiac disease, were within normal limits. Her total IgE level was 184 kU/L, with specific IgE tests for cypress, house dust mites (Dermatophagoides pteronyssinus and farinae), dog dander, timothy grass, olive, and wall pellitory being negative. ALEX (Allergy Xplorer) test results were negative, and skin prick tests for inhalants, cocoa, tomato, pork, mussels, peach, LTP, and profilin were negative. Quantitative and qualitative measurements of C1INH were within the normal range. Despite adherence to a nickel avoidance strategy, she continued to experience angioedema episodes, which appear not related to direct nickel contact or substances identified in patch testing. Then, she required further evaluation at our Pediatric Allergology and Immunology Unit for additional insights. This prompted further investigation into less obvious nickel exposure sources, leading to her discovering her orthodontic device. Consultation with her orthodontist confirmed that the orthodontic archwires and brackets contained the nickel-titanium (NiTi) alloy. After the removal of the orthodontic device, she presented a last episode of labial angioedema, which resolved in a few hours without taking any drugs. Complete improvement without treatment was observed in 2 weeks, and no recurrence was observed at 3 and 6 months of patient follow-up.

## Discussion and conclusions

This case underscores the importance of considering all potential sources of allergen exposure, even those that may not be immediately obvious, such as orthodontic devices, in patients with persistent symptoms despite adherence to known allergen avoidance strategies. The resolution of angioedema symptoms following the removal of the orthodontic device underscores the role of nickel as a trigger, blurring the lines between direct immunological mechanisms and non-immunological mast cell activation by nickel. Nickel sensitization caused by orthodontic treatments has been studied [5]. Although we did not assess our patient’s serum and saliva nickel levels, it’s critical to acknowledge that these concentrations may rise following the application of orthodontic devices [6]. The release of nickel into the intraoral cavity may be caused by saliva corroding the dental devices (brackets, bands, mesh, pads, and arches). Some studies discussed the phenomena of corrosion and wear of nickel in orthodontic devices. Studies on the corrosion of Ni-containing orthodontic alloys elucidate how in vivo exposure affects these archwires’ electrochemical properties and wear rates [7]​​​​. The protective nature of oral deposits on in vivo exposed NiTi and stainless steel archwires signifies a lesser corrosive impact than new devices. Yet, an increased wear and friction coefficient was noted, particularly in NiTi wires [7]​​. In orthodontic treatment, key processes such as galvanic corrosion, metal ion release, and surface degradation of nickel-containing appliances significantly influence their interaction with the oral environment and patient health, as highlighted by studies on fluoride mouthwashes, salivary concentrations of metals over 12 weeks, and galvanic interactions between dental alloys​ [8–11].

**Fig. 2:**
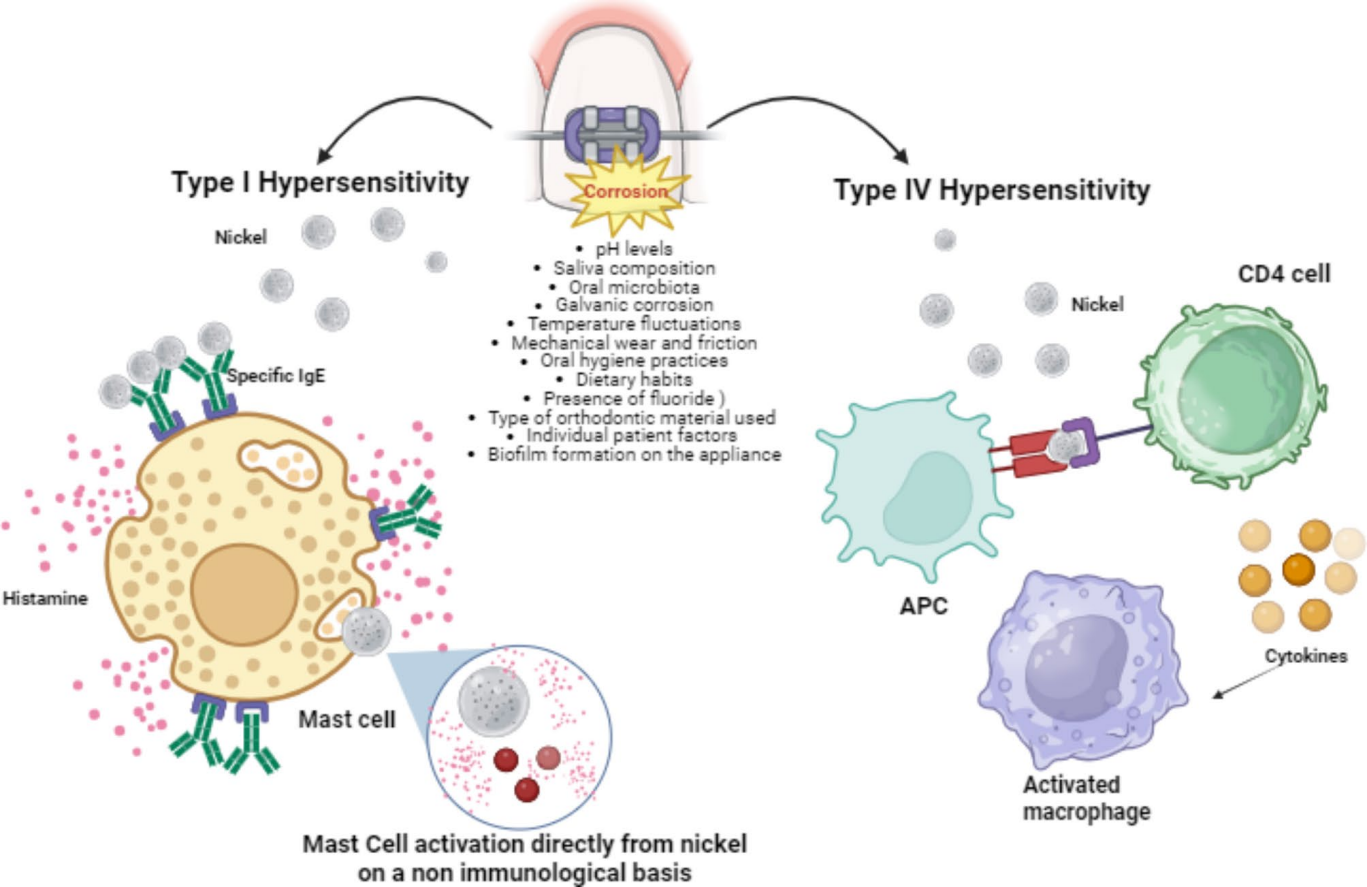
This image depicts an orthodontic appliance damaged by corrosion, outlining various contributing factors and the nickel’s release. On the left, nickel triggers Type I hypersensitivity reactions or directly initiates mast cell degranulation. On the right, the mechanism of a Type IV allergic response is illustrated. APC: antigen-presenting cell

Furthermore, as discussed in the comprehensive review on Ni leaching and its biological implications​​, the metallurgical aspects and intraorally reactions underpin our understanding of the potential for increased nickel levels post orthodontic appliance application [12]. This phenomenon, compounded by the invasive nature of appliance removal, may enhance nickel absorption by the oral mucosa, thereby justifying the observed clinical manifestations even after the device’s removal. This underlines that removing the dental device should also be done in total safety in such patients. The oral mucosa’s unique attributes, such as its rich vascularization and non-keratinized epithelium, make it a particular area for allergen absorption, possibly explaining systemic symptoms such as respiratory wheezing observed in some cases. The predominant symptom will result in labial angioedema due, however, to a mechanism of contact, as demonstrated in other case reports [12]. The differentiation between Type I and Type IV hypersensitivity reactions to nickel **(**Fig. 2) and the potential for nickel to directly activate mast cells on a non-immunological basis highlights the complexity of diagnosing and managing angioedema and contact dermatitis. However, we do not execute prick tests for nickel; this kind of angioedema could be oriented towards type I hypersensitivity, as reported in a case series of patients with contact urticaria to nickel [13]. Although it is possible to measure specific IgE antibodies against nickel, as demonstrated by the detection of specific IgE to nickel-conjugated human serum albumin (Ni-HSA) and nickel-conjugated exchange resin (Ni-resin) in patients with hard metal asthma [14], we were unfortunately unable to perform these tests in our case. In this case clinical history and symptom correlation remain key. However, other studies have shown the co-presence of Type I and IV hypersensitivity to nickel [15]. It has been postulated that nickel can act as an activator of mast cells on a non-immunological basis [16]: the release of histamine is triggered directly by the nickel ions without the involvement of IgE. These reactions mimic true IgE-mediated responses, they occur via different pathways. This could explain the occurrence of symptoms like angioedema in patients where IgE to nickel is not detectable, but mast cell degranulation still occurs. In Type IV hypersensitivity nickel ions come into contact with the skin, they act as haptens, forming a new antigenic structure: this model is recognized as foreign by the immune system. Langerhans cells in the skin, capture this hapten-protein complex; then they process and present the complex to CD4^+^ T cells in the lymph nodes, initiating a cascade of immune activation. During the sensitization, these T cells become specific to the nickel antigen. When the individual is re-exposed to nickel, these memory T cells are rapidly recruited to the site of contact and after activation, they release various cytokines, including IFN-γ and TNF-α, which are central to the Th1 response and mediate the inflammatory reaction. The Th17, also plays a role, producing IL-17, which helps recruit neutrophils to the site, sustaining inflammation and contributing to the tissue damage associated with allergic contact dermatitis. The hallmark of Type IV hypersensitivity is the chronic inflammation driven by these immune cells and cytokines, which manifests clinically as redness, itching, swelling, and vesicle formation at the site of contact. The clinical presentation of angioedema that we observed could potentially be interpreted as a form of mucositis. While angioedema typically involves the swelling of deeper layers of skin and mucosa, in this case, the swelling might represent a contact mucositis induced by direct contact with the metal apparatus: the device was exclusively touching the mucosal surfaces, leading to a localized inflammatory reaction. Although clinically similar to angioedema in its appearance, the underlying process may be more in line with mucosal irritation or inflammation, hence introducing the term “contact mucositis” to better describe this phenomenon. This inflammatory response the complexity of nickel-induced hypersensitivity.

While Type IV hypersensitivity is the most recognized mechanism of nickel allergy, it’s essential to consider that nickel can also induce Type I hypersensitivity reactions, although this is rare. In such cases, the immunological mechanism shifts from T-cell mediated to IgE-mediated, resulting in mast cell degranulation and immediate hypersensitivity reactions, such as urticaria and angioedema.

In expanding this understanding, it becomes crucial to recognize the genetic predispositions that may make certain individuals more susceptible to nickel allergy. For example, certain HLA alleles have been associated with increased sensitivity to nickel, particularly in individuals with repeated exposure, whether through jewelry, implants, or occupational contact [17]. In our case, there was a strong family history of nickel allergy: unfortunately, one of the limitations in this study was the inability to perform HLA typing in both the patient and her family. Other reactions related to nickel allergy include a range of both localized and systemic manifestations. Beyond the classic presentation of allergic contact dermatitis, individuals sensitized to nickel may experience respiratory symptoms, such as allergic rhinitis or asthma-like symptoms, particularly in occupational settings where nickel exposure occurs through inhalation [14, 18]. Additionally, oral allergy symptoms, such as swelling of the lips or oral mucosa (mucositis), can occur after contact with or ingestion of nickel-containing foods or materials [19]. Ocular symptoms, such as itching or swelling around the eyes, are often observed, particularly with the use of certain cosmetics containing nickel: these reactions can occur due to the direct contact of nickel-containing products triggering a localized allergic response [20].

Systemic Nickel Allergy Syndrome (SNAS) is a broader and more complex manifestation of nickel allergy [21]. This condition involves systemic symptoms following the ingestion of nickel through food, water, or environmental exposure. SNAS is characterized by gastrointestinal discomfort (such as nausea, bloating, and abdominal pain), generalized eczema, fatigue, and headaches. The mechanism of SNAS likely involves a combination of Type I and Type IV hypersensitivity reactions, along with systemic absorption of nickel that triggers widespread immune responses, contributing to the diverse range of symptoms. This concept challenges the idea that localized symptoms are purely driven by a single type of hypersensitivity. The braces release small amounts of nickel, which could be absorbed into the systemic circulation. This provides a plausible explanation for the complex reaction observed, going beyond the simple contact of nickel with the oral mucosa. Thus, the complexity of nickel-induced reactions, particularly in individuals with a high degree of sensitivity, requires us to consider that what presents as a local reaction may, in fact, involve systemic absorption and a combination of immune responses, extending the implications of nickel exposure beyond the site of contact. SNAS, along with other systemic and localized reactions, underscores the complexity of nickel allergy and the broad spectrum of clinical manifestations that can result from exposure to this metal allergen.

The implications of this case extend beyond clinical diagnosis to include considerations in dental and orthodontic material selection, underscoring the importance of screening for metal allergies before device placement and the potential benefits of alternative materials free from common allergens like nickel [22].

This case emphasizes the necessity of considering all potential sources of allergen exposure, particularly in environments as intricate as the oral cavity, where the combination of saliva, microbial presence, and dietary acids can accelerate metal corrosion and increase allergen release. Moreover, the differentiation between Type I and Type IV hypersensitivity reactions to nickel and the potential for nickel to directly activate mast cells on a non-immunological basis highlights the complexity of diagnosing and managing angioedema and contact dermatitis cases. The implications of this case extend beyond clinical diagnosis to include considerations in dental and orthodontic material selection, underscoring the importance of screening for metal allergies before device placement and the potential benefits of alternative materials free from common allergens like nickel.

## Data Availability

All clinical data and material are available in our service: Pediatric Allergology and Immunology of Policlinico Umberto I, Rome.

## Abbreviations

ACE: Angiotensin-converting-enzyme
AE: Angioedema
ALEX: Allergy Xplorer
APC: Antigen-presenting cells
C1INH: C1 Inhibitor
CT: Contact Dermatitis
HAE: Hereditary Angioedema
HS3ST: Heparan sulfate-glucosamine 3-O-sulfotransferase 6 gene
IFN-γ: Interferon gamma
IgE: Immunoglobulin E
IL-2: Interleukin 2
IL-4: Interleukin 4
IL-5: Interleukin 5
IL-17: Interleukin 17
LTP: Lipid Transfer Protein
Ni: Nickel
Ni-HSA: nickel human serum albumin
Ni-resin: Nickel-conjugated exchange resin
NiTi: nickel-titanium
TNF-α: Tumor Necrosis Factor Alpha
