# Correction: Nickel-induced labial angioedema in a pediatric patient with orthodontic braces: a case report

**DOI:** 10.1186/s13052-025-01923-x

**Published:** 2025-03-24

**Authors:** Fabrizio Leone, Alessandra Gori, Bianca Laura Cinicola, Giorgio Colletti, Elia Pignataro, Martina Capponi, Giulia Brindisi, Caterina Anania, Anna Maria Zicari

**Affiliations:** 1 Department of Maternal Infantile and Urological Sciences, Sapienza University of Rome, Rome, 00161 Italy; 2 Department of Translational and Precision Medicine, Sapienza University of Rome, Rome, 00161 Italy; 3 Department of Experimental Medicine, Sapienza University of Rome, Rome, 00161 Italy

**Correction: Italian Journal of Pediatrics (2024) 51:2**

10.1186/s13052-024-01833-4

The original article has been updated to correct a typo in co-author, Giorgio Colletti’s name, and to update the email address for co-author, Martina Capponi.

